# Cannabidiol Modulates Emotional Function and Brain-Derived Neurotrophic Factor Expression in Middle-Aged Female Rats Exposed to Social Isolation

**DOI:** 10.3390/ijms242015492

**Published:** 2023-10-23

**Authors:** Nadya Saad, Danielle Raviv, Tomer Mizrachi Zer-Aviv, Irit Akirav

**Affiliations:** 1 Department of Psychology, School of Psychological Sciences, University of Haifa, Haifa 3498838, Israel; nadyasaad295@gmail.com (N.S.); danielleraviv2@gmail.com (D.R.); tomer.mizrachi6@gmail.com (T.M.Z.-A.); 2 The Integrated Brain and Behavior Research Center (IBBRC), University of Haifa, Haifa 3498838, Israel

**Keywords:** middle-aged, depression, social isolation, BDNF, cannabidiol, females

## Abstract

Aging is associated with changes in cognitive and emotional function. Cannabidiol (CBD) has been reported to attenuate stress and anxiety in human and animal studies. In this study, we aimed to assess the therapeutic potential of CBD among middle-aged female rats exposed to social isolation (SI) and the potential involvement of brain-derived neurotrophic factor (BDNF) in these effects. Thirteen-month-old female rats were group-housed (GH) or exposed to social isolation (SI) and treated with vehicle or CBD (10 mg/kg). CBD restored the SI-induced immobility in the forced swim test and the SI-induced decrease in the expression of BDNF protein levels in the nucleus accumbens (NAc). CBD also increased the time that rats spent in the center in an open field, improved spatial training, and increased BDNF expression in the medial prefrontal cortex (mPFC) and basolateral amygdala (BLA). BDNF expression was found to be correlated with an antidepressant (in the NAc) and an anxiolytic (in the mPFC, BLA, NAc) phenotype, and with learning improvement in the PFC. Together, our results suggest that CBD may serve as a beneficial agent for wellbeing in old age and may help with age-related cognitive decline.

## 1. Introduction

Aging is a multifactorial process accompanied by extensive risk factors, such as increased susceptibility to chronic diseases, homeostatic dysregulation, and neurodegeneration [1]. Aging is associated with deterioration in memory processes [2] and emotional changes, specifically late-life depression [3,4].

Research has demonstrated cognitive impairments in middle-aged rats. For example, a longitudinal study demonstrated progressive spatial memory impairment in 12–13-month-old male rats [5]. Male rats in five different age groups (3, 12, 18, 24, and 30 months) exhibited impaired place navigation, which developed progressively with age and correlated with the nerve growth factor receptor (NGFr) cell count [6]. Compared to young rats, 13–14-month-old female rats demonstrated deficits in the visual attention task due to their comparative attentional load [7].

Middle-aged rats also exhibited differences in emotional reactivity compared to young rats, as manifested in depression- and anxiety-like behaviors. For example, 12-month-old male rats showed decreased crossings in the open field test, decreased time spent in the arms of the plus T maze test, and increased immobility in the forced swim test [8], and 24-month-old male rats exhibited a tendency to withdraw from social contacts [9].

Loneliness during aging due to the loss of close relatives or a lack of quality social relationships is known to enhance the likelihood of developing emotional dysfunction and rapid cognitive decline. Indeed, loneliness increases negativity and depressive symptoms and affects wellbeing and health status [10,11,12,13]. Rats are social mammals with a strong need for social contact [14]. Studies have demonstrated that social isolation (SI)—i.e., the deprivation of social relationships with cage mates—constitutes a chronic stress paradigm that exerts dramatic effects on behavioral performance involving neural, hormonal, cellular, and genetic mechanisms [15,16]. Hence, SI serves as a valid model for depression [17].

Although there are not many data on the effects of SI in middle-aged rats, studies have demonstrated severe impairments in emotional and cognitive functions [18,19,20,21]. For example, eight weeks of isolation among 10-month-old male rats drastically disrupted brain processes, as manifested in a decreased number of dendritic spines in the prefrontal cortex (PFC) and in the dorsal and ventral hippocampus, as well as decreased levels of brain-derived neurotrophic factor (BDNF) in the dorsal hippocampus [19]. Furthermore, six weeks of SI in 8-month-old male rats resulted in tau hyperphosphorylation in the hippocampus [18], suggesting impaired synaptic plasticity in regions associated with cognition. Four weeks of SI in 15-month-old male rats inhibited 5-HT expression in the dorsal rape [21]. It is important to note that stress paradigms in general, and SI specifically, appear to induce sex-dependent behavioral and biological effects in rats [22,23,24].

BDNF is a major regulator in cellular processes involved in the development and maintenance of normal brain functioning [25], particularly cell survival, cell growth and differentiation, and synaptic plasticity [25,26]. Decreased levels of BDNF protein have been found in schizophrenia and in bipolar and major depression disorder (MDD) patients [27] and correlated with cognitive dysfunction among individuals with late-life depression [27]. Postpartum studies have reported similar results in the PFC and the hippocampus of suicidal individuals exhibiting early-life depression [27]. Stress paradigms in rats have resulted in decreased BDNF expression in the hippocampus [28,29] and PFC [30]. Blocking the BDNF TrkB receptor in the basolateral amygdala (BLA) impaired fear extinction [31]. The downregulation of BDNF levels in the CA1 sub-region of the hippocampus was correlated with responses in rats that resembled post-traumatic stress disorder (PTSD) [32]. Eight weeks of SI in 10-month-old male rats decreased BDNF levels in the dorsal hippocampus [19]. Seventeen-month-old male rats exposed to four weeks of SI demonstrated decreased expression of the BDNF gene [33]. Another study indicated that 12 weeks of SI downregulated hippocampal BDNF expression was associated with anxiety- and depression-like behavior [34].

The effects of stressors and SI on BDNF levels are sex-dependent [35]. Among juvenile rats, one month of SI elevated BDNF levels in the hippocampus of female rats compared to males [22]. Chronic stress with foot shock decreased BDNF expression in the prelimbic PFC of adult female rats but had no effect in males [36], and chronic unpredictable mild stress decreased BDNF expression in adult female rats but not in males [37]. Not only is BDNF a biomarker for emotionally related brain deficits; it may also act as a bio-marker for the effective treatment of mood disorders in both human and animal studies [38]. Treatment with antidepressants or antipsychotics prevented/reversed the effects of stress on BDNF [39]. The injection of BDNF in the hippocampus decreased depression-like behavior, while the deletion of TrkB in the dentate gyrus or the inhibition of TrkB signaling blocked the effects of antidepressants on behavior [25]. The suggested mechanism is that BDNF functions as a modulator of the 5-HT system and vice versa, acting as a link between the antidepressant drug and the neuroplastic changes [25].

Cannabidiol (CBD), the second-most-prevalent active ingredient in the cannabis plant, was found to show anxiolytic, antidepressant, and antipsychotic properties in humans and animals [40] without the potential for abuse or dependence and without the typical spectrum of side effects common after treatment with Δ-9-tetrahydrocannabinol (THC) (e.g., dizziness, euphoria/high, thinking abnormalities, concentration difficulties, nausea, and tachycardia) [41,42]. It is important to note that CBD is not toxic and does not induce changes in food intake, catalepsy, heart rate, blood pressure, body temperature, or psychomotor functions [40,43].

Research has suggested that the antidepressant effects of CBD may be associated with alterations in the BDNF protein [44,45]. Acute and chronic CBD (10 mg/kg) treatment increased BDNF and synaptophysin mRNA in the medial PFC and hippocampus and produced antidepressant-like behaviors in 2-month-old male rats exposed to the FST stress paradigm [46]. We found in a previous study that this dose of CBD (i.e., 10 mg/kg) resulted in antidepressant effects in a rat model for depression [47]. The injection of the TrkB antagonist K252a eliminated the antidepressant behavioral effects of CBD [46]. In another study, both acute and chronic treatment with CBD (30 mg/kg) exhibited antidepressant-like effects in the FST and increased BDNF levels in the amygdala [48].

Middle-aged rats may demonstrate cognitive and emotional dysfunction, which is exacerbated by exposure to SI. Relatively few studies have focused on evaluating emotional behavior in old rats [9], and many of the studies assessing social stress paradigms are restricted to male rodents so as to avoid changes affected by circulating gonadal hormones and changes in sexual receptivity [35,49]. To bridge this gap in knowledge, in this study, we sought to examine the impact of chronic treatment with CBD on cognitive and emotional function in middle-aged female rats that were exposed to SI, as well as the involvement of BDNF.

## 2. Results

### 2.1. The Effects of CBD and SI on Behavior

#### 2.1.1. Open Field Test (OFT)

**Figure 1:**
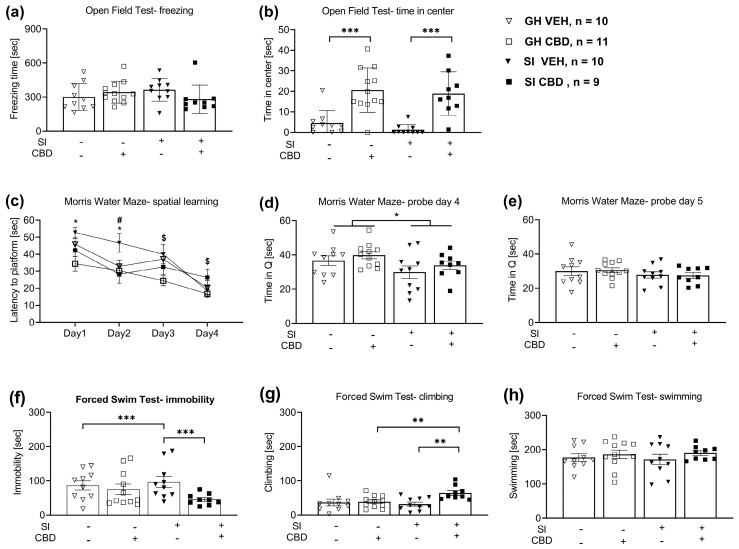
The effects of SI and CBD on cognitive and emotional behavior. (**a**) No differences were observed in freezing time in the open field. (**b**) CBD females spent more time at the center of the open field than vehicle females did. (**c**) In the Morris water maze, CBD rats demonstrated decreased latency compared to vehicle rats on day 1 and day 2 (for the SI condition) and on day 3 (for the GH condition). Moreover, GH CBD rats demonstrated decreased latency compared to SI CBD rats on day 4, and GH vehicle rats demonstrated decreased latency compared to SI vehicle females on day 2. (**d**) SI rats spent less time in the platform quadrant on the probe test on day 4 than GH rats did. (**e**) No differences were observed in the probe test on day 5. (**f**) In the FST, SI vehicle rats demonstrated increased immobility compared to GH vehicle and SI CBD rats. (**g**) SI CBD rats demonstrated increased climbing compared to SI vehicle and GH CBD rats. (**h**) No differences were observed in the swimming time. * *p* < 0.05; **, *p* < 0.01; ***, *p* < 0.001. For (**c**): * *p* < 0.05 indicates a difference between SI-CBD and SI-Veh on days 1, 2; # *p* < 0.05 indicates a difference between GH-Veh and SI-Veh on day 2; $ *p* < 0.05 indicates a difference between GH-CBD and GH-Veh (day 3) and CBD-SI (day 4).

For freezing in the open field, two-way ANOVA [drug *×* SI; 2 *×* 2] revealed no significant effects (F_(1,37)_ = 3.24, *p* = 0.08) (Figure 1a).

For the time in the center, two-way ANOVA [drug × SI; 2 × 2] revealed a significant drug effect (F_(1,37)_ = 41.43, *p* < 0.001), suggesting that CBD increased the amount of time that the rats spent in the center compared to the vehicle group rats (Figure 1b).

#### 2.1.2. Morris Water Maze (MWM)

For the latency to reach the hidden platform, repeated measure analysis showed a significant effect for training days (F_(3,108)_ = 30.169, *p* < 0.001), drug (F_(1,36)_ = 9.95, *p* < 0.01), SI (F_(1,36)_ = 5.89, *p* < 0.05), training days *×* drug interaction (F_(3,108)_ = 2.80, *p* < 0.05), and training days *×* drug *×* SI interaction (F_(3,108)_ = 2.9, *p* < 0.05). Post hoc comparisons during the training days showed that the SI CBD rats demonstrated decreased latency compared to the SI vehicle rats on day 1 and day 2 (*p* < 0.05). Moreover, the SI vehicle rats demonstrated increased latency compared to the group-housed (GH) vehicle rats on day 2 (*p* < 0.05). GH CBD rats demonstrated decreased latency compared to GH vehicle rats on day 3 (*p* < 0.01) and compared to CBD SI rats on day 4 (*p* < 0.05) (Figure 1c).

For the probe test on day 4, two-way ANOVA revealed a significant main effect for SI (F_(1,36)_ = 5.08, *p* < 0.05), such that SI rats spent less time in the platform quadrant compared to GH rats (Figure 1d). No significant effects were found on probe day 5 (F_(3,36)_ = 0.674, *p* < 0.57) (Figure 1e).

#### 2.1.3. Forced Swim Test (FST)

For immobility, two-way ANOVA [drug × SI; 2 × 2] revealed a significant effect for drug (F_(1,36)_ = 26.3, *p* < 0.001), SI (F_(1,36)_ = 4.365, *p* < 0.05), and drug × SI interaction (F_(1,36)_ = 18.46, *p* < 0.001). Post-hoc analysis revealed that the SI vehicle rats demonstrated increased immobility compared to the GH vehicle rats (*p* < 0.001). In addition, the SI CBD rats demonstrated decreased immobility compared to the SI vehicle rats (*p* < 0.001) (Figure 1f).

For climbing, two-way ANOVA [drug × SI; 2 × 2] revealed a significant effect for drug (F_(1,36)_ = 6.0, *p* < 0.05) and for drug × SI interaction (F_(1,36)_ = 4.65, *p* < 0.05). Post-hoc analysis revealed that the SI CBD rats demonstrated increased climbing compared to the SI vehicle rats (*p* < 0.01). In addition, the SI CBD rats demonstrated increased climbing compared to the GH CBD rats (*p* < 0.01) (Figure 1g).

For swimming, two-way ANOVA [Drug *×* SI; 2 *×* 2] did not reveal significant effects (F_(3,36)_ = 0.479, *p* = 0.699) (Figure 1h).

### 2.2. The Effects of CBD and SI on BDNF Protein Expression

#### 2.2.1. Medial PFC (mPFC)

**Figure 2:**
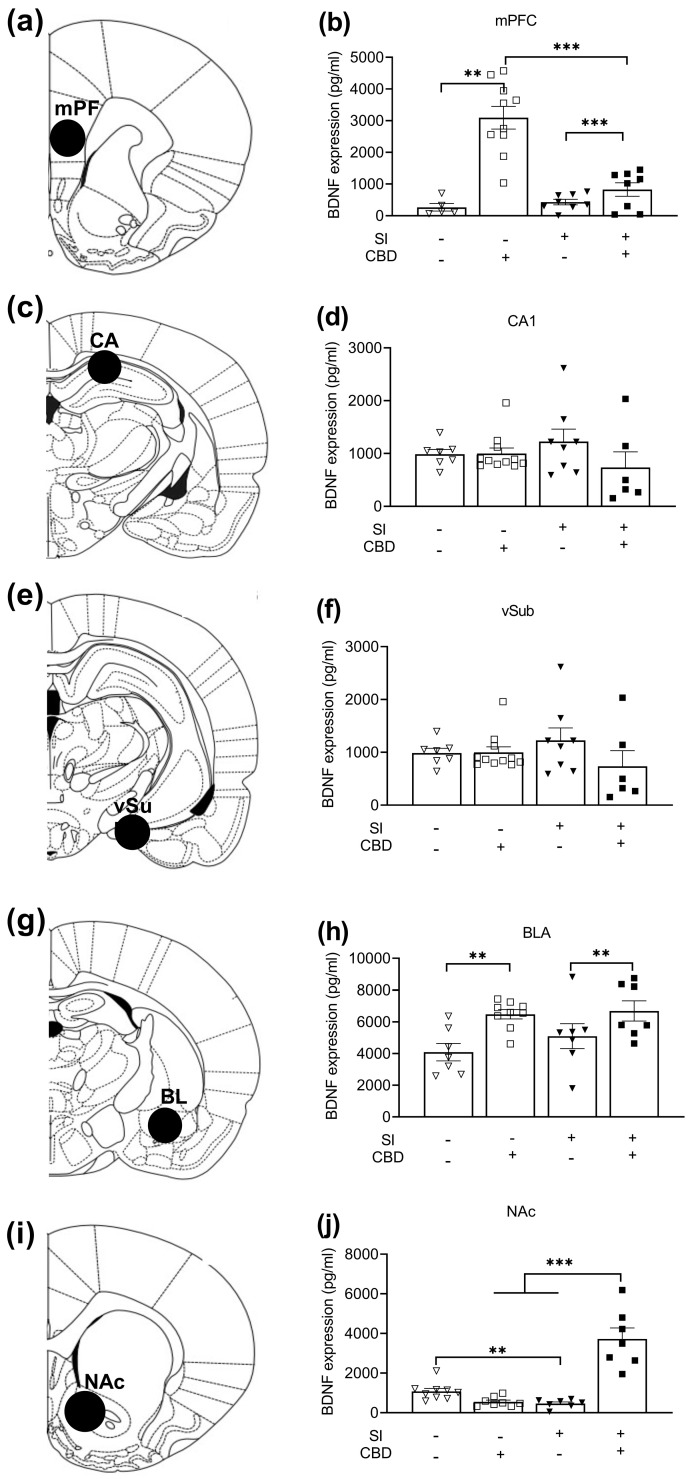
The effects of SI and CBD on BDNF expression: (**a**) rat brain atlas illustration indicating mPFC punch location. (**b**) CBD females demonstrated increased BDNF expression compared to SI and GH vehicle rats. Moreover, GH CBD rats demonstrated increased BDNF expression compared to SI CBD rats. (**c**) Rat brain atlas illustration indicating CA1 punch location. (**d**) No differences were observed in the CA1. (**e**) Rat brain atlas illustration indicating VSUB punch location. (**f**) No differences were observed in the VSUB. (**g**) Rat brain atlas illustration indicating BLA punch location. (**h**) CBD females demonstrated increased BDNF expression in the BLA compared to vehicle rats. (**i**) Rat brain atlas illustration indicating NAc punch location. (**j**) SI vehicle females demonstrated decreased BDNF expression compared to GH vehicle rats, and SI CBD females showed increased BDNF expression compared to SI vehicle and GH CBD rats. ** *p* < 0.01, *** *p* < 0.001.

Two-way ANOVA [Drug × SI; 2 × 2] on BDNF expression in the mPFC (Figure 2b) revealed significant effects of SI (F_(1,24)_ = 9.54, *p* < 0.01), drug (F_(1,24)_ = 35.42, *p* < 0.001), and drug × SI interaction (F_(1,24)_ = 14.99, *p* < 0.001). Post hoc analysis revealed increased BDNF expression in CBD rats compared to vehicle rats in both the SI and the GH conditions (*p* < 0.001; *p* < 0.01; respectively). Moreover, GH CBD rats demonstrated increased BDNF expression compared to SI CBD rats (*p* < 0.001).

#### 2.2.2. CA1

Two-way ANOVA [drug × SI; 2 × 2] on BDNF expression in the CA1 (Figure 2d) did not reveal any significant effects (F_(1,28)_ = 1.06, n.s).

#### 2.2.3. Ventral Subiculum (VSUB)

Two-way ANOVA [drug × SI; 2 × 2] on BDNF expression in the VSUB (Figure 2f) did not reveal any significant effects (F_(1,30)_ = 0.16, *p* = ns).

#### 2.2.4. Basolateral Amygdala (BLA)

Two-way ANOVA [drug × SI; 2 × 2] on BDNF expression in the BLA (Figure 2h) revealed a significant drug effect (F_(1,26)_ =12.27, *p* = <0.01), such that CBD rats demonstrated increased BDNF expression compared to vehicle rats.

#### 2.2.5. Nucleus Accumbens (NAc)

Two-way ANOVA [drug × SI; 2 × 2] on BDNF expression in the NAc (Figure 2j) revealed significant effects for drug (F_(1,27)_ = 25.96, *p* = <0.0001), SI (F_(1,27)_ = 22.6, *p* = <0.0001), and drug × SI interaction (F_(1,27)_ = 49.63, *p* = <0.001). Post hoc analysis revealed decreased BDNF expression in the SI vehicle rats compared to the GH vehicle rats (*p* < 0.01), and increased BDNF expression in the SI CBD rats compared to the SI vehicle (*p* < 0.001) and GH CBD rats (*p* < 0.001).

Atlas illustrations of a coronal view of brain areas for molecular analysis are shown in Figure 2a (PFC), Figure 2c (CA1), Figure 2e (VSUB), Figure 2g (BLA), and Figure 2i (NAc).

### 2.3. Correlations between BDNF Levels and Behavior

**Figure 3:**
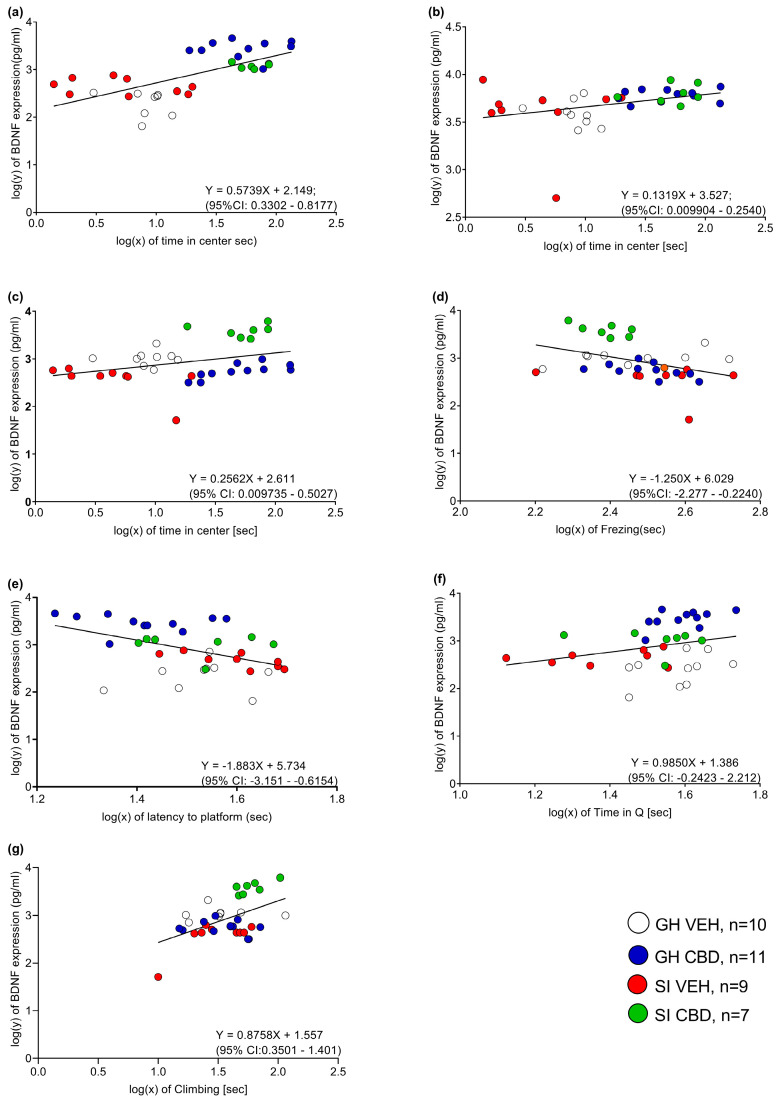
Scatter plots of the BDNF expression index (log-transformed) and behavioral performance index (log-transformed) (regression line in solid dark). (**a**) Time spent in the center and BDNF levels in the mPFC. (**b**) Time spent in the center and BDNF levels in the BLA. (**c**) Time spent in the center and BDNF levels in the NAc. (**d**) Freezing levels and BDNF levels in the NAc. (**e**) Latency to reach the hidden platform and BDNF levels in the mPFC. (**f**) Time spent in the platform quadrant (Q) and BDNF levels in the mPFC. (**g**) Climbing levels and BDNF levels in the NAc.

To explore the association between the anxiogenic- and depressive-like phenotype of the rats and their BDNF expression, Pearson bivariate correlation tests (see Table 1) were conducted between the expression of BDNF in all brain regions tested (mPFC, CA1, VSUB, BLA, NAc) and the rats’ behavioral performance. See Figure 3 for scatter plots of the BDNF expression and behavioral performance.

Significant correlations were found between the time spent in the center and the BDNF levels in the mPFC (r = 0.422, *p* < 0.05), BLA (r = 0.433, *p* < 0.05), and NAc (r = 0.0431, *p* < 0.05), suggesting that increased BDNF expression in these brain regions is associated with an anxiolytic phenotype.

Freezing levels were negatively correlated with BDNF levels in the NAc (r = −0.395, *p* < 0.05), and climbing levels were positively correlated with BDNF levels in the NAc (r = 0.724, *p* < 0.001), suggesting that increased BDNF expression in this brain region is associated with an anxiolytic (i.e., decreased freezing) and antidepressant (i.e., increased climbing) phenotype.

Moreover, BDNF levels in the mPFC were negatively correlated with the average latency to reach the hidden platform of the four training days (r = −0.522, *p* < 0.01) and with the time spent in the platform quadrant on probe test 4 (r = 0.437, *p* = 0.02), suggesting that increased PFC BDNF expression is associated with better learning of the task and better retrieval.

## 3. Discussion

The results of our study point to therapeutic properties for CBD in middle-aged female rats. CBD induced anxiolytic- and antidepressant-like effects in the open field and forced swim tests and improved spatial learning and memory in the water maze. CBD increased BDNF expression in the mPFC and BLA and restored an SI-induced decrease in NAc BDNF. Moreover, significant correlations were found between increased BDNF expression in the PFC, BLA, and NAc and an anxiolytic phenotype. Increased NAc BDNF expression was correlated with an antidepressant effect, and a better performance in the memory test was correlated with PFC BDNF expression.

Exposure to SI increased immobility in the FST in middle-aged female rats, with no effect on freezing or the time in the center in the open field, further suggesting that SI is associated with depression-like behavior [50] and not anxiety [51,52,53,54]. Regardless of SI exposure, CBD was found to have both antidepressant- and anxiolytic-like effects, in line with previous studies in males [3,55,56,57,58,59] and females [55,60,61,62]. For example, CBD decreased anxiety-like behavior in the elevated plus maze [60] and decreased freezing behavior and prevented fear reinstatement in a contextual fear paradigm in adult (3–4 months) female rats [62]. In a genetic rat model of depression, adult females and males treated with 30 mg/kg CBD demonstrated pro-hedonic behavior in the saccharin preference test, increased exploration in the novel object recognition test, and decreased immobility in the FST [55].

CBD females also spent more time in the quadrant in which the platform was previously located in the probe test on day 4, and their learning curve in the spatial task was better than that of the vehicle rats. This suggests that CBD does not impair memory and may even improve it. It is possible that this better performance stems from the antidepressant/anxiolytic effects of CBD. In the probe tests, each rat went through one trial in the maze with the platform removed. This could explain why we did not observe a significant difference in the second probe test on day 5, as the rats learned that the platform’s previous location was no longer relevant.

CBD increased BDNF expression in the mPFC and BLA and restored an SI-induced decrease in BDNF in the NAc, in line with previous findings [46,48,63]. CBD was shown to increase BDNF levels in the BLA and to have antidepressant behavioral effects [48]. Moreover, CBD exposure upregulated BDNF in the mPFC, while repeated exposure increased BDNF in the striatum, with a slight decrease in the mPFC, suggesting a CBD dose-dependent and anatomically specific modulation of BDNF [63].

The better performance on the memory test on the last day of training was significantly correlated with PFC BDNF expression. The mPFC is important for short-term spatial working memory [64] and in overall goal-directed activity during spatial navigation [65].

The affective fronto-limbic circuity includes multiple cortical and limbic structures, with the PFC, BLA, and NAc serving as the main regions associated with emotional processing. Specifically, BLA and NAc are known to mediate anhedonia, anxiety, and reduced motivation in depression disorder [66].

Nevertheless, the reports about the effects of BDNF on depression and anxiety are controversial, as increased BDNF levels were shown to produce a anxiety-like or depression-like phenotype [66,67]. For example, research has demonstrated that blocking BDNF in the NAc has an antidepressant-like effect, whereas microinjecting BDNF into the NAc results in a depression-like effect in the FST [68]. Moreover, several studies have demonstrated an increase in BDNF mRNA expression in the BLA after exposure to different types of stress (intermittent water immersion stress, restraint, fear conditioning), suggesting specific regional protein involvement with anxiety- or depression-like behaviors [69,70,71]. Some studies have also shown an increase in the BDNF protein of mRNA expressions in the PFC following stress [72,73].

Research has suggested that BDNF protein alterations in those regions are highly dependent on the stress exposure period. Specifically, an increase in BDNF expression occurs after short periods of stress, whereas longer periods will eventually lead to a decrease [74,75].

We found no significant alterations in the expression of BDNF in the CA1 or ventral subiculum areas of the hippocampus following SI or CBD administration. Previous studies suggested that BDNF downregulation in the hippocampus is dependent mainly on the period of SI; for example, BDNF expression in the hippocampus decreased after 10 weeks of SI, but not after 6 weeks [76].

Another possible explanation for the discrepancy regarding the role of BDNF in depression involves serotonergic- and dopaminergic-dependent mechanisms; for example, in a different depression model, olfactory bulbectomy, endogenous BDNF levels increase in an attempt to counteract the bulbectomy-induced loss in serotonergic function in the PFC and hippocampus [77]. In the social defeat stress model of depression, BDNF was shown to be required for the development of social aversion using the viral-mediated, mesolimbic-dopamine-pathway-specific knockdown of BDNF [68].

Previous studies have demonstrated alterations in BDNF protein expression and behavioral phenotypes following stress paradigms such as SI in middle-aged rats [18,19,33,34]. Nonetheless, most research has been restricted to male rodents to avoid research noise due to hormonal changes [35,49]. To the best of our knowledge, this is the first study to address BDNF alterations in middle-aged female rats exposed to SI stress and the potential therapeutic effect of CBD. Taken together, our findings suggest that CBD may have beneficial effects on cognition and emotion in middle-aged females and that these effects involve BDNF activation.

## 4. Materials and Methods

### 4.1. Subjects

Middle-aged female Sprague Dawley (SD) rats (13 ± 1 months old, ~360 g, Envigo, Jerusalem, Israel) were group-housed (3 per cage; 59 × 28 × 20 cm) or isolated at 22 ± 2 °C under 12 h light/dark cycles (lights turned on at 07:00). Rats were allowed water and laboratory rodent chow ad libitum.

### 4.2. Drug Treatment

Over a period of one month, rats were injected daily with vehicle or CBD (10 mg/kg, i.p.). CBD was dissolved in 2% Tween-80 and 98% saline, freshly prepared and administered in 1 mL/kg of vehicle. The CBD dose was based on other studies [47,78,79]. See supplementary information (SI) for the effects of CBD on weekly weight gain; Table S1.

### 4.3. Social Isolation Stress Paradigm (SI)

Half of the rats were randomly selected to be exposed to social isolation (SI) for 8 weeks. The SI rats were individually housed in plastic cages and given food and water ad libitum [19,80], while the other half were group-housed (GH).

### 4.4. Behavioral Tests

All rats were exposed to the same battery of behavioral tests. The tests were carried out in the following order: anxiety-like behavior in the novel open field arena test, spatial learning and memory in the Morris water maze test, and despair-like behavior in the FST. Tests were separated by a 24 h period and took place between 09:00 and 16:00 under dim lighting (15–20 lx).

#### 4.4.1. Open Field (OFT)

The open field consists of a closed wooden box (72 × 72 cm). The walls and the floor are painted black and placed under dim red light (<10 lux). Rats were placed in the open field arena for testing. The arena was thoroughly cleaned between each trial with ethanol 10%. The rats’ movements were recorded and analyzed for 5 min using a video tracking system (Ethovision × T 14.0, Noldus Information Technology, Wageningen, The Netherlands) to measure anxiety-like behavior. The time spent in the arena center and the time spent freezing were calculated.

#### 4.4.2. Morris Water Maze Test (MWM)

The Morris water maze (MWM) is used to test spatial learning and memory. The maze consists of a black circular tank (150 cm in diameter) filled with water (23 ± 1) °C to a depth of 30 cm. The maze was divided into four equal quadrants, and a transparent escape platform (12 cm in diameter) was placed in a constant position in the middle of the northeastern quadrant, 2–3 cm below the water surface. Rats were exposed to three learning trials each day for four days. Each trial started with the placing of the rat into a random quadrant in the maze, facing the wall. Each trial was terminated automatically as soon as the rat reached the platform or when 60 s had elapsed. The rat was allowed to stay on the platform for 5 s. Rats that did not find the platform within 60 s were guided to the platform and placed there for 10 s. After each trial, rats were gently dried with a towel and returned to the home cage for 3 min. The latencies and swimming paths for the rats to search for the platform were monitored using a video tracking system (Ethovision × T 14.0, Noldus Information Technology). A probe test was conducted at the end of the fourth day (to assess working memory) and on the fifth day (to assess long-term memory). In the probe test, each rat went through one trial in the maze with the platform removed. Animals were allowed to swim freely for 60 s while the time and distance in each quadrant were recorded.

#### 4.4.3. Forced Swim Test (FST)

Each rat was forced to swim for 15 min inside an acrylic cylinder (34 cm of water at 23 °C). After 24 h in the home cage, the rat was put back in the cylinder and forced to swim for 5 min. The amounts of time spent climbing, swimming (active coping measures), and immobile (a passive coping measure) were recorded and analyzed manually to assess depressive-like behavior in rodents. This is based on the assumption that when an animal is placed in a container filled with water, it will first make efforts to escape but will eventually exhibit immobility that may be considered to reflect a measure of behavioral despair [50].

### 4.5. BDNF Protein Measurement via Enzyme-Linked Immunosorbent Assay (ELISA)

Brains were extracted and frozen in liquid nitrogen within 5 min of decapitation and stored at −80 °C until dissection. The basolateral amygdala (BLA), medial prefrontal cortex (PFC), nucleus accumbens (NAc), CA1, and ventral subiculum (VSUB) areas of the hippocampus were punched out using a 0.5 mm puncher. The punch location was verified using the rat brain atlas [81]. ELISA was performed according to the manufacturer’s instructions with the Rat BDNF ELISA Kit (ab213899, Abcam, Cambridge, MA, USA). Plates were incubated with BDNF antibodies overnight at 4 °C. After non-specific binding was blocked with a blocking buffer, the test samples were added. A second specific antibody was incubated to bind the captured BDNF. Plates were incubated with chromogenic substrate, and substrate absorbance fluorescence was recorded at 450 nm on an ELISA plate reader. All samples were assayed in duplicate.

### 4.6. Experimental Design

**Scheme 1:**
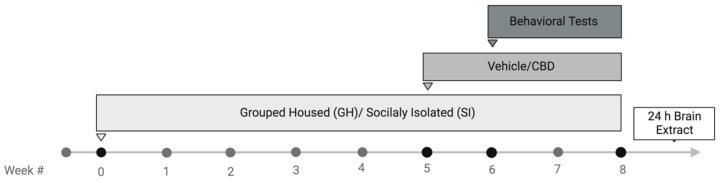
Study design. Middle-aged female rats were randomly group-housed (GH) or socially isolated (SI) for a total of eight weeks. At the beginning of the fifth week, they were injected daily with vehicle or cannabidiol (CBD; 10 mg/kg, i.p.) until the end of the experiment. During the last two weeks, a battery of behavioral tests was conducted: the open field (day 42), the Morris water maze (days 45–49), and the forced swim test (days 55–56). All rats were exposed to the same battery of behavioral tests. The tests occur under dim lighting (15–20 lx) and take place between 9:00 and 13:00 h. Twenty-four hours after the last behavioral test (day 57), brains were removed for ELISA analysis of BDNF in the PFC, ventral subiculum, CA1, NAc, and BLA. The sample size was based on previous studies in our lab using a stress model and similar behavioral tests in rats [82].

The following Scheme 1 shows the study design.

### 4.7. Statistical Analysis

The results are expressed as means ± SEM. For statistical analysis, two-way ANOVA, mixed design two-way ANOVA, *t*-tests, and Pearson bivariate correlation tests were conducted as indicated. In linear regression analysis, we used adjusted *p* values for multivariable analysis [values style: 0.1234 (ns); 0.0332 (*); 0.0021 (***); 0.0002 (***); <0.0001 (****)]; the mean significance was calculated as the deviation from zero; log (y) and log (x) transformations were applied for better data presentation and normality of distributions. Data were analyzed using SPSS 27 (IBM, Chicago, IL, USA) and GraphPad 8.0.2 (Prsim, Boston, MA, USA). The homogeneity of variance was confirmed with Levene’s test for equality of variances. The normality assumption was examined using the Shapiro–Wilk test (*p* < 0.05).

## Figures and Tables

**Table 1:** Pearson correlation coefficients between BDNF expression and behavior.

	mPFC (*n* = 28)	CA1 (*n* = 32)	VSUB (*n* = 34)	BLA (*n* = 25)	NAc (*n* = 25)
OFT—time in center	r = 0.422 *p* = 0.025	r = −0.152 *p* = 0.406	r = −0.172 *p* = 0.330	r = 0.433 *p* = 0.027	r = 0.431 *p* = 0.032
OFT—freezing	r = 0.075 *p* = 0.73	r = −0.183 *p* = 0.316	r = 0.089 *p* = 0.617	r = −0.214 *p* = 0.305	r = −0.395 *p* = 0.051
MWM test—training days average	r = −0.522 *p* = 0.004	r = −0.101 *p* = 0.581	r = −0.063 *p* = 0.723	r = −0.230 *p* = 0.269	r = −0.135 *p* = 0.519
MWM probe (day 4)	r = 0.437 *p* = 0.02	r = 0.105 *p* = 0.567	r = −0.123 *p* = 0.489	r = −0.001 *p* = 0.996	r = −0.257 *p* = 0.215
MWM probe (day 5)	r = 0.353 *p* = 0.065	r = 0.027 *p* = 0.881	r = 0.046 *p* = 0.796	r = 0.222 *p* = 0.286	r = 0.030 *p* = 0.887
FST—immobility	r = 0.272 *p* = 0.161	r = 0.005 *p* = 0.980	r = 0.071 *p* = 0.692	r = −0.046 *p* = 0.828	r = −0.383 *p* = 0.059
FST—climbing	r = −0.001 *p* = 0.995	r = −0.14 *p* = 0.941	r = 0.136 *p* = 0.443	r = 0.391 *p* = 0.053	r = 0.724 *p* = <0.001
FST—swimming	r = −0.325 *p* = 0.092	r = 0.004 *p* = 0.981	r = −0.155 *p* = 0.380	r = −0.187 *p* = 0.370	r = −0.004 *p* = 0.984

mPFC—medial prefrontal cortex; VSUB—ventral subiculum; BLA—basolateral amygdala; NAc—nucleus accumbens; OFT—open field test; MWM—Morris water maze; FST—forced swim test.

## Data Availability

Not applicable.

## Supplementary Materials

The following supporting information can be downloaded at: https://www.mdpi.com/article/10.3390/ijms242015492/s1.
