# Supplementary material for: Cannabidiol Modulates Emotional Function and Brain-Derived Neurotrophic Factor Expression in Middle-Aged Female Rats Exposed to Social Isolation
Source: Int J Mol Sci. 2023 Oct 23;24(20):15492. doi: 10.3390/ijms242015492 (PMC10607116; doi:10.3390/ijms242015492)
Supplement: Supplementary file 1 [file ijms-24-15492-s001.zip › ijms-2630308-supplementary.pdf]

Supplementary Materials

Table S1. The effects of CBD on weekly weight gain. For weights taken in the last 4 weeks of the experiment (during CBD injections), repeated measures ANOVA [Drug×SI×Days; 2×2×4] revealed a significant effect of Days ($F_{(3,108)}$= 7.894, P<.001) suggesting that all groups significantly gained weight throughout this period. No effects were found for SI ($F_{(1,36)}$ =0.822, NS), drug ($F_{(1,36)}$ =0.401, NS), or SI × drug interaction ($F_{(1,36)}$= 0.834, NS).

Groups	Week 1	Week 2	Week 3	Week 4
**SI VEH, N=10**				
1	335	330	328	319
2	325	322	326	330
3	366	360	351	365
4	360	355	345	341
5	342	383	378	372
6	316	310	320	327
7	330	350	343	332
8	312	306	314	318
9	314	320	385	387
10	320	333	324	330
**GH VEH, N=10**				
1	400	380	380	385
2	300	305	300	301
3	300	295	291	292
4	310	305	303	310
5	300	290	291	285
6	333	335	335	338
7	339	341	342	340
8	321	325	330	331
9	322	330	331	335
10	360	355	344	370
**SI CBD, N=9**				
1	339	343	342	347
2	347	342	351	355
3	315	327	333	338
4	314	323	330	331
5	342	339	343	345
6	334	341	339	349
7	333	332	327	337
8	340	341	343	345
9	315	323	327	338

**GH CBD, N=11**				
1	325	323	328	331
2	311	328	325	339
3	332	339	344	344
4	370	365	361	374
5	335	363	346	341
6	339	347	345	345
7	323	327	336	350
8	317	325	339	351
9	330	338	330	339
10	328	335	335	338
11	307	319	327	339
